# Molecular and physiological manifestations and measurement of aging in humans

**DOI:** 10.1111/acel.12601

**Published:** 2017-05-23

**Authors:** Sadiya S. Khan, Benjamin D. Singer, Douglas E. Vaughan

**Affiliations:** ^1^ Department of Medicine Northwestern University Feinberg School of Medicine Chicago IL 60611 USA

**Keywords:** aging, biological age, biomarkers, score, senescence

## Abstract

Biological aging is associated with a reduction in the reparative and regenerative potential in tissues and organs. This reduction manifests as a decreased physiological reserve in response to stress (termed homeostenosis) and a time‐dependent failure of complex molecular mechanisms that cumulatively create disorder. Aging inevitably occurs with time in all organisms and emerges on a molecular, cellular, organ, and organismal level with genetic, epigenetic, and environmental modulators. Individuals with the same chronological age exhibit differential trajectories of age‐related decline, and it follows that we should assess biological age distinctly from chronological age. In this review, we outline mechanisms of aging with attention to well‐described molecular and cellular hallmarks and discuss physiological changes of aging at the organ‐system level. We suggest methods to measure aging with attention to both molecular biology (e.g., telomere length and epigenetic marks) and physiological function (e.g., lung function and echocardiographic measurements). Finally, we propose a framework to integrate these molecular and physiological data into a composite score that measures biological aging in humans. Understanding the molecular and physiological phenomena that drive the complex and multifactorial processes underlying the variable pace of biological aging in humans will inform how researchers assess and investigate health and disease over the life course. This composite biological age score could be of use to researchers seeking to characterize normal, accelerated, and exceptionally successful aging as well as to assess the effect of interventions aimed at modulating human aging.

*‘*Time is but the stream I go a‐fishing in. I drink at it; but while I drink I see the sandy bottom and detect how shallow it is. Its thin current slides away, but eternity remains’. – Henry David Thoreau, Walden
.

## Introduction

The number of individuals aged 60 or older will increase dramatically in the next three decades. As the fastest growing age‐strata worldwide, the global population over 60 will surpass two billion by 2050: a 12‐fold increase from 1950 (United Nations Department of Economic and Social Affairs Population Division 2013). In the 20th century, decreased mortality and lengthening of average human lifespan shifted the worldwide demographic structure toward the aged. This shift stemmed initially from treatment of infectious diseases and subsequently cardiovascular disorders (Fries, 2005). However, an increase in late‐life disability has accompanied gains in healthy years lived (health span) and longevity (Crimmins *et al*., 1994). Age represents the primary risk factor for chronic diseases, including cardiovascular, malignant, and neurodegenerative conditions. Extremely aged individuals who survive in good health to the end of the human lifespan are rare, and a fixed limit to human lifespan may exist (Dong *et al*., 2016).

**Figure 1:**
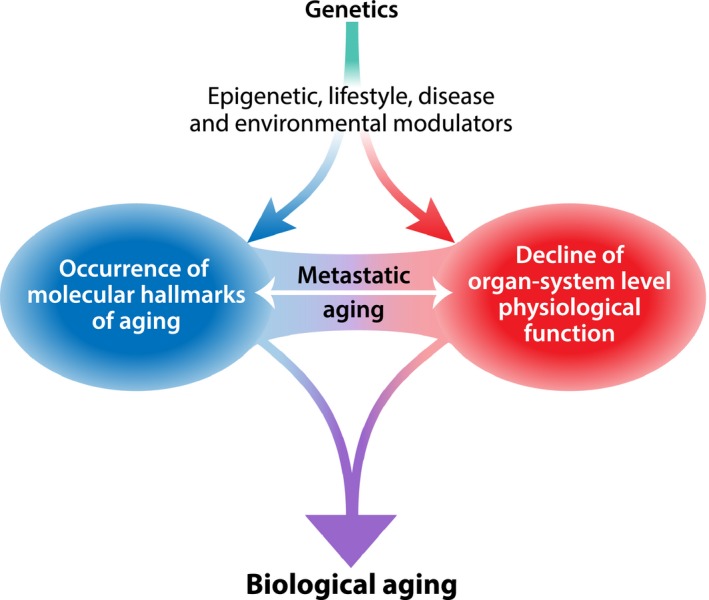
Biological aging is a multifactorial process. The molecular hallmarks of aging and organ‐specific physiological function are both influenced by genetic, epigenetic, and environmental factors. Metastatic aging may contribute to differential aging in remote tissues through a paracrine mechanism.

Biological aging is associated with a reduction in the reparative and regenerative potential in tissues and organs. This reduction manifests as decreased physiological reserve in response to stress (termed *homeostenosis*) and a time‐dependent failure of complex molecular mechanisms that cumulatively create disorder. Aging inevitably occurs with time in all organisms and emerges on a molecular, cellular, organ, and organismal level with genetic, epigenetic, and environmental modulators (Fig. 1). Individuals with the same chronological age and their organs exhibit differential trajectories of age‐related decline, and it follows that we should assess biological age distinctly from chronological age. Understanding the molecular and physiological phenomena that drive the complex and multifactorial processes underlying biological aging in humans will inform how researchers assess and investigate health and disease over the life course. In this review, we outline mechanisms of aging with attention to well‐described molecular and cellular hallmarks, discuss normal human aging at the organ‐system level, suggest methods to measure biological age, and propose a framework to integrate molecular and physiological data into a composite score that measures biological aging in humans.

## Search strategy and selection criteria

Findings for this review were identified by searches of MEDLINE, Current Contents, PubMed, and references from relevant articles using the search terms ‘aging’, ‘measurement’, and ‘assessment’. Abstracts and reports from meetings were included only when they related directly to previously published work. With exceptions for historical interest, only articles published in English between 1980 and 2016 were included.

## Molecular mechanisms of aging

**Figure 2:**
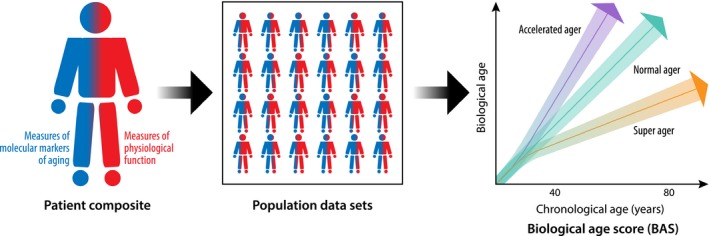
Conceptual derivation of a biological age score (BAS) that combines molecular markers derived from measures of the molecular hallmarks of aging (represented here in blue, e.g., telomere length and gene‐specific DNA methylation) and measures of physiological function (represented here in red, e.g., FEV_1_ and e’ velocity) that are longitudinally assessed throughout the life course. Potential mathematical modeling approaches for integrating individual components into a composite BAS include multiple linear regression, principal component analysis, and Klemera and Doubal's method with derivation and validation in population‐based data sets. The BAS graph depicts three hypothetical aging trajectories: 1) normal ager, 2) super ager, and 3) accelerated ager with colored areas representing confidence intervals and demonstrating overlap at young ages. The aging lines are depicted hypothetically as straight lines, but the trajectory of biological aging is not known.

The heritable contribution to aging is limited for most humans, with genetics accounting for only 20–30% of lifespan variability in human twin and founder population family studies (Mitchell *et al*., 2001; Kenyon, 2010). However, heritable factors may represent a significantly larger contribution to lifespan at *extreme ages*, and the exceptionally aged may offer an opportunity to find rare genetic variants associated with longevity (Tan *et al*., 2010; Sebastiani *et al*., 2012). Regardless, no single factor or molecular mechanism explains progressive age‐related homeostenosis. Inter‐related molecular and cellular phenomena occur during normal aging, intensify during accelerated or premature aging, and can be mitigated to increase lifespan. López‐Otín and colleagues proposed nine so‐called hallmarks of aging—genomic instability, telomere attrition, epigenetic alterations, loss of proteostasis, deregulated nutrient sensing, mitochondrial dysfunction, cellular senescence, stem cell exhaustion, and altered intercellular communication—that frame mechanisms underlying senescence (Lopez‐Otin *et al*., 2013). As we discuss in this section, many hallmarks suggest potential therapeutic targets to restore age‐associated functional decline and homeostenosis, although potential therapies are not completely benign. Translation of these hallmarks with surrogate measurements are important to include in a composite biological age score (BAS) because of their direct relationship with the molecular basis of aging (Fig. 2).

Mouse and human data implicate genomic instability in accelerated aging (Burtner & Kennedy, 2010) with myriad exogenous agents (e.g., radiation and xenobiotic compounds) as well as endogenous processes (e.g., DNA replication errors and reactive oxygen species [ROS]) causing damage to DNA (Hoeijmakers, 2009). Cumulative genomic damage disturbs homeostasis and impacts health span (Moskalev *et al*., 2013). Genome maintenance represents a potential therapeutic target, as augmenting mitotic regulators involved with chromosomal segregation such as BubR1 improves health span in mice (Baker *et al*., 2013). Although malignant transformation as a consequence of genomic manipulation remains a concern, high‐level expression of BubR1 reduced tumorigenesis in transgenic mice.

Telomeres, the chromatin tips responsible for preventing degradation at chromosomal ends, shorten and become increasingly susceptible to damage with age (Blackburn *et al*., 2006). Most mammalian somatic cells lack telomerase—a specialized DNA polymerase responsible for repairing telomeres after cell division—which results in replication‐dependent sequence loss at chromosomal ends, often leading to replicative senescence (the Hayflick limit) (Hayflick & Moorhead, 1961; Jiang *et al*., 2008). Accordingly, telomerase overexpression increases median lifespan in mice (Bernardes de Jesus *et al*., 2012). However, *in vivo* evidence of the causal role telomerase plays in aging remains controversial, as telomerase knockout mice demonstrate no overt phenotype and reduced longevity only after multiple generations (Rudolph *et al*., 1999). In humans, deficiencies in the telomerase complex cause a variety of age‐related pathologies, including prematurely gray hair, pulmonary fibrosis, liver disease, and aplastic anemia (Armanios & Blackburn, 2012). Gene therapy encoding telomerase components may hold promise to halt or even reverse telomere shortening in humans (Ramunas *et al*., 2015). However, gene therapy remains in its infancy with ongoing concerns regarding oncogenesis (Hacein‐Bey‐Abina *et al*., 2003).

Epigenetic alterations—heritable changes in phenotype that are independent of DNA sequence mutations—occur with aging and impact cellular function (Sen *et al*., 2016). Overall, global heterochromatin loss and redistribution occur with an increase in transcriptional noise (Bahar *et al*., 2006; Tsurumi & Li, 2012). Specifically, H4K16 acetylation and H4K20 and H3K4 trimethylation increase, while H3K9 methylation and H3K27 trimethylation decrease with age (Han & Brunet, 2012). Further, deleting the H3K4 and H3K27 methylation complexes extends lifespan in worms and flies (Greer *et al*., 2010; Siebold *et al*., 2010). The role DNA methylation at cytosine‐phospho‐guanine islands plays in aging remains less clear, with age‐associated global gene‐activating hypomethylation but gene‐repressive hypermethylation at tumor suppressor gene loci (Maegawa *et al*., 2010). Large‐scale genomewide DNA methylation‐based epigenetic analyses using a variety of cell types and tissues have identified an ‘epigenetic clock’ that is closely correlated with healthy aging (Horvath, 2013). Unlike cumulative DNA damage and many other hallmarks of aging, epigenetic alterations are ostensibly reversible and embody promising pharmacologic targets for therapies designed to promote healthy aging. DNA methyltransferase and histone deacetylate inhibitors represent two potential drug classes (So *et al*., 2011; Wang *et al*., 2013), although significant refinement in enzyme isoform specificity will be required to limit off‐target effects of these compounds including immunomodulation (Wang *et al*., 2015).

Protein homeostasis (proteostasis) becomes impaired with aging and enhanced proteostasis can maintain the integrity of the proteome and delay mammalian senescence (Zhang & Cuervo, 2008; Koga *et al*., 2011). The two key proteolytic pathways responsible for protein quality control—the autophagy–lysosomal system and the ubiquitin–proteasome system—become hypofunctional with aging (Calamini *et al*., 2012; Tomaru *et al*., 2012). Therapies aimed at promoting healthy aging could target the proteostasis system (Calamini *et al*., 2012), as deubiquitylase inhibitors and proteasome activators augment clearance of harmful protein in human cells (Lee *et al*., 2010). Of note, the proteasome activator characterized by Lee *et al*. displayed dose‐dependent and drug target‐independent toxicity (hypoproliferation) in cultured cells, suggesting that further pharmacologic refinement of proteasome activators may be needed.

Deregulated nutrient sensing represents an important and potentially druggable hallmark of aging, as anabolic signaling causes accelerated aging and caloric restriction extends lifespan in murine and nonhuman primate models (Colman *et al*., 2014; López‐Otín *et al*., 2016; Vermeij *et al*., 2016). Nutrient sensing systems, including the amino acid‐sensing mammalian target of rapamycin (mTOR) as well as low‐energy state detectors (AMP kinase and the sirtuins), contribute to the aging process (Houtkooper *et al*., 2010). The sirtuin family of NAD‐dependent protein deacetylases may promote healthy aging in yeast, flies, and mice (Houtkooper *et al*., 2012), and compounds that raise NAD^+^ levels may restore lost mitochondrial function and promote longevity (Gomes *et al*., 2013). In addition, the insulin and insulin‐like growth factor 1 (IGF‐1) signaling (IIS) pathway, which targets the FOXO transcription factors and mTOR complexes, represents an extremely well‐conserved pro‐aging pathway (Kenyon, 2010; Barzilai *et al*., 2012). Mutations that reduce the level or function of growth hormone, the IGF‐1 receptor, or intracellular pathway components including AKT, mTOR, and FOXO closely associate with longevity in model organisms as well as humans (Arum *et al*., 2014). However, aging mouse models display *decreased* IIS activity, which raises the possibility that IIS activity represents a defensive response to systemic damage (Schumacher *et al*., 2008). Strikingly, mice fed the mTOR inhibitor rapamycin in late life experienced extension of median and maximal lifespan, supporting a role for mTOR signaling and pharmacologic inhibition in lifespan regulation (Harrison *et al*., 2009). The long‐term effects of rapamycin administration, including sex‐dependent differences in outcomes, remain incompletely defined (Fischer *et al*., 2015).

Mitochondrial dysfunction in the form of decreased respiratory chain efficiency, resulting electron leak, and diminished ATP production may contribute to senescence (Green *et al*., 2011). A significant body of data supports ROS as a cause of accelerated aging (Harman, 1965). However, recent evidence suggests that limited oxidative stress may actually be beneficial to health span (Hekimi *et al*., 2011) and that mitochondrial ROS impart advantageous effects on healthy cellular function (Sena & Chandel, 2012) up to a threshold (Hekimi *et al*., 2011). Compelling evidence demonstrates that therapies designed to improve fitness could exploit mitohormesis—the concept that repeated low‐level toxic exposures can trigger a beneficial compensatory mitochondrial response that ultimately leads to augmentation in cellular fitness (Haigis & Yankner, 2010).

With aging, there exists a propensity for stable cell cycle arrest known as cellular senescence (Hayflick & Moorhead, 1961; Collado *et al*., 2007). DNA damage (independent of telomeres) and derepression of the *INK4/ARF* locus induce cellular senescence and occur with advancing age. *INK4/ARF* locus expression of p16^INK4a^ and p19^ARF^ acts as a cellular checkpoint that critically prevents propagation of damaged and possibly malignant cells. As a biomarker, protein levels of p16^INK4a^ and p19^ARF^ correlate with chronological age remarkably well in humans and model organisms with a large difference (up to an order of magnitude) comparing young and old tissues (Krishnamurthy *et al*., 2004; Ressler *et al*., 2006). The correlation holds for almost all tissues examined. However, from a functional perspective, accumulation of senescent cells may ultimately become deleterious when regenerative capacity grows exhausted and progenitors cannot replace senescent cells. For example, stem cell attrition occurs in multiple tissues with aging, and DNA damage, p16^INK4a^ expression, and telomere shortening cause decreased hematopoietic stem cell proliferation (Sharpless & DePinho, 2007). Therapeutic removal of p16^INK4a^‐positive cells could extend healthy lifespan (Baker *et al*., 2016).

Senescent cells display a characteristic secretory profile—the senescence‐associated secretory phenotype—that contributes to low‐level systemic inflammation (so‐called inflammaging) and likely facilitates the spread of pro‐senescence signals through tissues and systemically alters the extracellular matrix in parallel (Kuilman *et al*., 2010). These factors enable a senescence messaging system (SMS) and include interleukins, IGFBP3, plasminogen activator inhibitor‐1 (PAI‐1), and transforming growth factor‐β (Kuilman & Peeper, 2009; Capell *et al*., 2016; Ozcan *et al*., 2016). In addition to paracrine signaling that results from the senescence‐associated secretory phenotype, accumulation of tissue damage and failure of the immune system to clear damaged proteins, pathogens, and compromised or malignant cells promote inflammaging (Senovilla *et al*., 2012). Metastatic aging—in which aging in one tissue accelerates aging in other tissues (Lavasani *et al*., 2012)—may occur via gap junctions and ROS signaling and via miRNAs secreted into the blood that promote senescence in remote tissues (Grillari & Grillari‐Voglauer, 2010; Nelson *et al*., 2012). Finally, heterochronic parabiosis and knockout murine experiments provide additional supportive evidence of a distinct proteomic signature of senescence with alterations in circulating systemic factors (e.g., GDF‐11, CCL11, Klotho, β2 microglobulin); however, emerging evidence questions the role GDF‐11 plays in aging mice (Conboy *et al*., 2005; Villeda *et al*., 2011; Eren *et al*., 2014; Laviano, 2014; Sinha *et al*., 2014; Brun & Rudnicki, 2015; Smith *et al*., 2015). Therapeutic administration or blockade of SMS components could ameliorate inflammaging in humans, although significant concerns persist regarding off‐target effects including skeletal and cardiac muscle wasting (Harper *et al*., 2016).

The interplay of the above mechanisms and pathways contribute to aging on an organismal level and offer potential parameters to include in a composite assessment of biological age as well as targets for therapies aimed to counter age‐related functional decline and morbidity. In addition to targeted interventions, understanding the molecular mechanisms underpinning senescence could translate into biomarkers of biological age (Table 1).

**Table 1:** Biomarkers of aging derived from the hallmarks of aging (Lopez‐Otin *et al*., 2013)

Hallmark of aging	Biomarkers
Telomere attrition	Blood leukocyte‐derived telomere length Telomere dysfunction‐induced proteins
Epigenetic alterations	H4K16 acetylation; H4K20 and H3 K4, K9, and K27 methylation DNA methylation patterns Noncoding RNA patterns (e.g., microRNA expression profiles)
Loss of proteostasis	Proteomics Amyloid‐β‐derived diffusible ligands (ADDLs)
Deregulated nutrient sensing	Insulin‐like growth factor‐1 (IGF‐1) Metabolomics
Mitochondrial dysfunction	Number of mitochondria Mitochondrial DNA copy number Mitochondrial protein levels
Cellular senescence and pro‐inflammatory cytokines (altered intercellular communication)	SMS: PAI‐1, IGFBP‐3, IL‐6, TGF‐β IL‐1β, TNF‐α p16^INK4a^ and p19^ARF^ p53, p21 Senescence‐associated β‐galactosidase (SABG) Youth‐associated (GDF‐11) and age‐associated (CCL11, Klotho, β2 microglobulin) circulating factors

## Organ‐level physiological changes of aging

**Figure 3:**
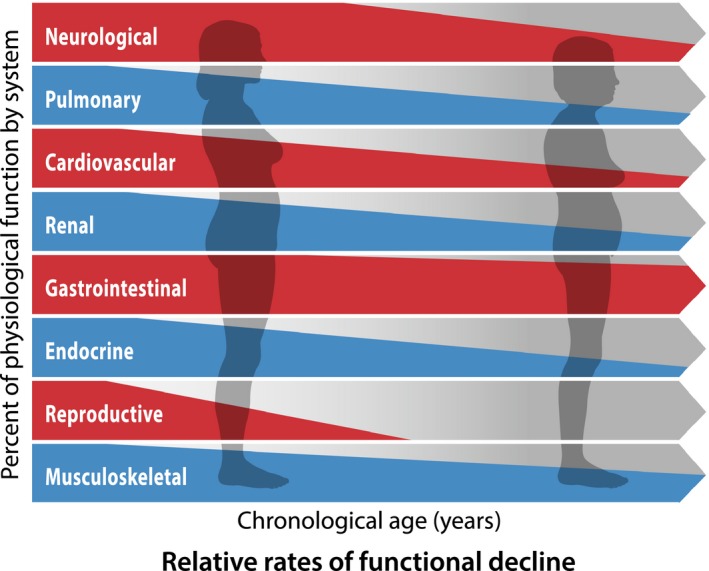
Relative rates of decline of organ‐specific physiological function. Different organ systems may carry a specific vulnerability to age (i.e., the cardiovascular system appears to suffer biological aging more rapidly than the gastrointestinal system).

Physiological aging involves a progressive detrimental change in maximal organ function with differential trajectories across organ systems (Fig. 3) (Shock, 1956). Importantly, multiple factors including genetics, environmental conditions, and developmental programming determine maximal organ function, which varies significantly between individuals (Lange *et al*., 2015). Aging affects all organ systems and must be assessed through a variety of physiological measures, as aging varies greatly organ‐to‐organ and person‐to‐person and results in impaired reserve capacity and limited ability to respond to stress. While there appears to be an organ‐specific or organ‐differential resilience and vulnerability of aging, *frailty* refers to the cumulative decline and increasing homeostatic imbalance that precedes the ultimate consequence of aging: death (Fries, 2005).

### Cardiovascular

The aging cardiovascular system displays decreased compliance of the aorta and large vessels (Vaitkevicius *et al*., 1993). This increased arterial stiffness results in a widened pulse pressure with raised systolic blood pressure (due to increased resistance to blood ejection from the left ventricle [LV]) and lowered diastolic pressure (due to a more rapid pressure decrease in diastole). Subsequent changes include increases in LV afterload, mass, wall thickness, and LV end‐diastolic volume. Further alterations in calcium influx cause reduced LV compliance and delayed LV relaxation or decreased diastolic function as assessed by Doppler echocardiography parameters (e.g., E‐wave/A‐wave velocity ratio, septal and lateral e’‐wave velocity depth) (Sun *et al*., 2004). Intrinsic heart rate declines due to both atrial pacemaker cellular loss (50–75% by age 50) and His bundle fibrosis (Cheitlin, 1989). Fibrosis and calcification occur at the aortic valve cusp bases, annular valvular rings, and fibrous trigones. Finally, aged individuals demonstrate decreased responsiveness to β‐adrenergic receptor stimulation in cardiomyocytes, decreased reactivity to baroreceptor and chemoreceptor output, and increased circulating catecholamines resulting in reduced exercise tolerance and decreased cardiac output (Davies *et al*., 1996). These changes increase the heart's vulnerability to development of age‐related cardiovascular pathology including hypertension, congestive heart failure, atrioventricular block, and aortic stenosis. Additionally, atherosclerosis is linked to premature biological aging with senescent cells identified in coronary artery disease plaques (Wang & Bennett, 2012).

### Pulmonary

Lung function represents one of the few consistently reliable physiological markers of aging. With advancing age, peak aerobic capacity falls with a greater than 20% decline per decade after age 70 (Fleg *et al*., 2005). The lungs lose elastic tissue, which causes decreased surface area available for gas exchange (increased anatomic dead space) as alveolar ducts enlarge (Gillooly & Lamb, 1993). Chest wall compliance decreases and dominates the increase in lung compliance; functional residual capacity decreases as a result of the fall in total respiratory system compliance. Forced vital capacity (FVC) declines 0.15‐0.30 L per decade in nonsmoking men, and the forced expiratory volume in one‐second (FEV_1_) falls 0.20–0.30 L/s per decade with a steeper decline in the 7th and 8th decades (Xu *et al*., 1995). Physiological restriction may result for some individuals, although population mean total lung capacity does not change significantly with age. Residual volume increases by about 10% per decade due to an increased closing volume: the lung volume at which small airways in dependent lung zones begin to collapse during exhalation. Ventilation–perfusion mismatching increases with age, as airways in the better‐perfused dependent lung zones have an increased likelihood of closure during exhalation. Diffusion capacity decreases around 5% per decade, although hypoxemia does not typically develop. Further, advancing age is associated with a diminished central drive to the respiratory muscles in response to hypoxemia, hypercapnia, and mechanical load; exercise training may attenuate this hyporesponsiveness. Collectively, the above changes in combination with decreased respiratory muscle strength and reduced efficacy of mucociliary clearance result in increased susceptibility to pneumonia (Enright *et al*., 1994).

### Renal

The kidneys develop a diffuse glomerulosclerosis with age (up to 30% by age 75) (Nyengaard & Bendtsen, 1992); remaining glomeruli display impaired filtering ability. Only 3% of donor kidneys from 18–29‐year‐olds and over 70% from 70 to 77‐year‐olds contain nephrosclerosis. Creatinine clearance falls 7.5–10 mL per decade on average with a large variance. Serum creatinine, however, may remain constant due to decreased production with age. Cystatin C may therefore represent a more accurate renal function marker in the elderly (Christensson & Elmstahl, 2011). Aged individuals maintain fluid and electrolyte balance in the absence of a significant challenge; however, stress can impair maximal diluting and concentrating ability in older individuals (Christensson & Elmstahl, 2011). In addition, the aging kidney demonstrates a decreased ability to acidify urine and excrete an acid load. Renal plasma flow decreases with age due in part to increased local vasodilating prostaglandin concentration (Ungar *et al*., 2000). The renal vascular system undergoes spiraling of the afferent arterioles and intimal fibrosis (Tracy *et al*., 1988) as well as shunt development between afferent and efferent arterioles.

### Immune and hematologic systems

The aging immune system displays progressive changes collectively described as immunosenescence; these changes result in increased susceptibility to infection, malignancy, and autoimmunity. The adaptive and innate immune systems both exhibit functional decline with aging, although innate immunity appears better preserved (Weiskopf *et al*., 2009). Pro‐B‐cell production declines with less striking changes in T‐cell precursors. Regulatory T cells lose their suppressive function (Tsaknaridis *et al*., 2003) and accumulate in visceral adipose tissue. Indeed, age‐associated chronic inflammation is associated with an inflammatory signature within visceral adipose tissue (Lumeng *et al*., 2011).

The aging immune system carries a greater likelihood of clonal expansion and hematologic malignancy (Jaiswal *et al*., 2014). Bone marrow mass decreases and undergoes fatty replacement with a resultant decrease in total bone marrow hematopoietic tissue (Geiger & Rudolph, 2009). This decrease in bone marrow mass leads to a loss of functional reserves, reduced hematopoiesis with hypoproliferative hematopoietic stem cells, and increased incidence of anemia and myeloid diseases. However, iron flux, red cell lifespan, total white blood cell count, and blood volume do not decline with age. Platelet responsiveness increases as do multiple coagulation factor levels (Franchini, 2006).

### Neurologic function

Cognitive decline with aging is multifactorial and related to changes in structure as well as synaptic plasticity. Cerebral tissue atrophy and diminished cerebral perfusion result in significant white matter loss, but neuronal dropout varies by brain region with little or no loss in some regions (Bertoni‐Freddari *et al*., 1996). In addition, dopaminergic signaling demonstrates a progressive decrease in signaling via the D2 receptor (Roth & Joseph, 1994). Functional MRI studies demonstrate less‐coordinated activation in brain regions focused on higher‐order cognitive functions, which suggests a global loss of integrative function with aging (Andrews‐Hanna *et al*., 2007). Gene expression profiling studies show that significant changes in synaptic gene expression contribute to altered higher‐order integration (Jiang *et al*., 2001). These alterations in synaptic plasticity and loss as well as impaired neurogenesis may predispose aged individuals to neurodegenerative disorders such as Alzheimer's disease and Parkinson's disease (Loerch *et al*., 2008).

### Other organ systems

Aging modifies the digestive, hepatic, and endocrine systems to varying degrees. The digestive system undergoes only modest changes with time, and normal aging does not cause malnourishment. Micronutrient absorption in the small intestine may decrease with age but not to a level that impairs homeostasis. Liver mass decreases 20–40% with age, and hepatic blood flow declines (Zoli *et al*., 1989). Serum albumin may fall slightly, but routine liver chemistries do not change with time (Rahmioglu *et al*., 2009). The aging liver displays decreased vitamin K‐dependent clotting factor synthesis (Froom *et al*., 2003). Alterations in metabolism influence lifespan in experimental models and potentially embody high‐yield translational targets. Insulin resistance and physiological declines in circulating insulin‐like growth factor characterize the aging process (Barzilai *et al*., 2012). Further, aging results in decreased β‐cell regeneration in pancreatic islets (Sartori *et al*., 2014). Metabolomics approaches have identified a potential longevity signature characterized by increased levels of circulating citric acid cycle intermediates (Cheng *et al*., 2015).

Finally, the musculoskeletal, integumentary, sensory, and behavioral systems undergo a multitude of changes with aging. Muscle mass and contractile force decrease and may limit mobility (Delbono, 2011). Age‐related loss of muscle mass (sarcopenia) occurs along with qualitative changes in muscle characterized by fat and connective tissue infiltration. Findings from the AGES‐Reykjavik study suggest that muscle composition may be associated with mortality risk (Reinders *et al*., 2015, 2016). Skin changes include epidermal thinning, decreased dermal elasticity, and diminution of subdermal fat that result in increased susceptibility to trauma and infection (Elewa *et al*., 2015). Progressive miosis, decreased corneal transparency, and increased lens rigidity cause presbyopia and decreased visual acuity (Salvi *et al*., 2006). Sensory cell loss and cochlear neuron dropout lead to presbycusis (Gates & Mills, 2005). Finally, healthy behavior change becomes less likely with age, seemingly as a result of alterations in social networks among older adults (Tucker *et al*., 2004).

## Comprehensive assessement of biological aging

Chronological age offers limited information regarding the complex processes driving biological aging. Individuals with the same chronological age vary greatly in health and in disease and disability prompting the utility of defining a ‘biological age’. The conceptualization of such a biological age distinct from chronological age has been proposed by many researchers with measures as crude as functional ‘frailty’ and as sophisticated as patterns of DNA methylation (Borkan & Norris, 1980; Ravindrarajah *et al*., 2013; Weidner *et al*., 2014; Marioni *et al*., 2015). While much research has focused on quantification of biological aging, a comprehensive and integrative score incorporating molecular biomarkers and physiological functional parameters is lacking. Current strategies to assess systemic biological age carry significant limitations as individual parameters to accurately reflect an individual's global homeostenosis have been elusive. Further, biological plausibility suggests that no single biomarker is likely to suffice given the underlying multisystem nature of the aging process with changes occurring on a molecular and organ‐based level underscoring the utility of an aggregate score of biological aging. Scoring systems require careful integration of molecular markers (surrogates of the hallmarks of aging) with longitudinal physiological functional measures, yet little consensus exists regarding optimal methods for creation and evaluation of a composite biological age score (BAS).

In human populations, identifying and characterizing successful agers who remain disease‐free at advanced age with physiological function significantly above their age cohort represent a promising approach to derive and validate a BAS (Fig. 2). Centenarians exemplify an exceptional survival phenotype who delay disability and disease until an average age of 93 years (Andersen *et al*., 2012). However, centenarians are rare, making it difficult to enroll meaningful sample sizes and answer important research questions. Moreover, longevity and healthy aging may not be synonymous, making lifespan a complicated phenotype to study in addition to the burden of cost and time using this approach. Data from the Leiden Longevity Study and the Long Life Family Study suggest that compression of morbidity into later years accelerates with the age of the cohort (Westendorp *et al*., 2009; Newman *et al*., 2011). The result is an oftentimes lengthy period of health before age‐associated morbidity develops at old age. Therefore, in any cohort, identifying subclinical disease can help ensure that data sets accurately classify successful aging, delayed morbidity, and increased longevity rather than age‐associated disease states (Fries, 2005).

Investigators have proposed composite scoring systems in epidemiologic studies such as the National Health and Nutrition Examination Survey (NHANES). The derivation cohort for the NHANES‐based measure of biological age contained more than 9000 participants aged 30–75 years at baseline and integrated physiological and biochemical markers at a single time point (Levine, 2013). The Dunedin Study birth cohort, which identified differences in the pace of aging in young adults over a decade, utilized the NHANES score (Belsky *et al*., 2015). The Healthy Aging Index in the Cardiovascular Health Study and the Modified Physiological Index in the Health, Aging, and Body Composition Study, represent additional examples of cohort‐derived scores that correlate with chronological age yet lack integration of existing novel markers derived from the molecular hallmarks of aging, which connote a true biological age (Ludwig & Smoke, 1980; Baker & Sprott, 1988). Several limitations of existing scores impede current investigations, including small sample sizes, limited testing of variables, omission of novel molecular markers, lack of participant‐level longitudinal follow‐up with repeated measures in the same individual, and dearth of population‐level replication and validation in clinical settings for predictive ability.

We propose a conceptual framework for a composite BAS, which integrates available molecular measurements based on the hallmarks of aging (Table 1) and functional organ physiology measurements (Table 2) across the life course. Comprehensive and repeated assessments over time of existing and emerging molecular biomarkers and organ‐specific functional measures in longitudinal epidemiologic cohorts in parallel with the use of sophisticated bioinformatics methodologies are needed to derive a global BAS. Conceptually, the BAS represents an integrated biomarker signature that assesses systemic aging on a population level (Cohen *et al*., 2015). Criteria for inclusion of molecular and physiological parameters into a composite score require that the individual parameters be independently associated with aging and provide additive information when combined. In general, components of the BAS should be i) highly correlated with chronological age, ii) predict organ‐system and global age‐related decline, and iii) be minimally invasive, readily observable, and reliably measured. Investigation into the optimal parameters used to derive a BAS will require collection and analysis of data sets that include successful agers without morbidity as well as accelerated agers with genetic progeroid syndromes, inflammatory pathologic conditions (e.g., human immunodeficiency virus, autoimmune disease states, chronic kidney disease), and disease‐related morbidity. Derivation and validation of a BAS will require multiple types of study designs including observational prospective population‐based cohorts, leveraging large sample sizes with repeated measures over several decades as well as case–control studies and family‐based studies to incorporate less common phenotypes of interest, including successful agers and accelerated agers with nongenetic and genetic conditions.

**Table 2:** Measures of organ‐specific changes in physiological function

Organ system	Measures of organ‐specific function
Cardiovascular	Brachial pulse pressure LV mass Relative wall thickness Echocardiographic parameters (E/A, e’) Pulse wave velocity Augmentation index Aortic valve calcification Heart rate variability
Respiratory	Peak aerobic capacity Spirometry (FEV_1_, FVC, FEV_1_/FVC ratio) Lung volumes (TLC, FRC, RV) DLCO Quantitative ventilation–perfusion scanning
Renal	Cystatin C Creatinine clearance
Immune	Immune risk profile (assessment of T‐cell proliferation in response to mitogens, B‐cell numbers, CD4:CD8 T‐cell ratio, and CMV serologic status)
Bone marrow	Hemoglobin
Neurocognitive	Mini‐mental status examination Cognitive battery Functional MRI
Digestive and hepatic	Vitamin K‐dependent clotting factor levels
Endocrine	Thyroid biochemical tests Fasting glucose Insulin Circulating estrogen and testosterone levels
Musculoskeletal	Hand grip strength Unipedal stance test of balance Grooved pegboard test of fine motor coordination SF‐36 physical functioning scale
Integumentary	Skin elasticity Thickness Wrinkle parameter
Sensory	Visual acuity Auditory test Retinal microvascular damage (arteriovenous ratio)

Optimal mathematical modeling using various methodologies such as multiple linear regression, principal component analysis, and Klemera and Doubal's method to derive scoring systems will require head‐to‐head comparisons (Takeda *et al*., 1982; Nakamura *et al*., 1988; Klemera & Doubal, 2006). The use of regression equations will be helpful in initially identifying individual components to be included in the BAS. However, given that aging is a systemic process composed of interdependent processes, redundancies in selected aging parameters may exist. The use of principal component analysis will be critical to determine the number of components or biomarkers to include to create the most parsimonious model and exclude overlap in contribution of molecular markers and physiological parameters. Standardizing the process of creating and evaluating longevity phenotypes with a BAS will accelerate research that endeavors to define healthy aging mechanisms, identify interventions to promote health span, and allow translation and validation of therapies that promote healthy aging.

## Future directions

Humans are mortal, and natural limits to lifespan will inevitably persist. While aging cannot be escaped (Gompertz, 1825), postponing senescent changes and disease onset offers the potential to extend fitness, vitality, and years lived free of morbidity and frailty. While there is evidence for modulation of lifespan in preclinical models and animal species via genetic and pharmacologic interventions, translation of these findings in human populations is needed. Here, we discuss the limited scientific database regarding mechanisms and physiological changes of aging and describe a framework to capture the complexity of the aging process in a novel integrative score of biological aging. A comprehensive view of biological aging is particularly germane at this time when randomized clinical trials are being discussed and planned with the intention of testing the therapeutic benefit of drugs such as metformin on human aging (Albert Einstein College of Medicine of Yeshiva University 2015; Longo *et al*., 2015). Integrating novel molecular assays derived from the hallmarks of aging and physiological measurements will help develop a composite BAS for use as a complex quantitative phenotype to translate mechanistic findings of biological pathways into humans. While conclusive methods to measure biological age remain debatable, deriving a BAS will serve multiple purposes: i) propel aging research at the molecular and cellular level, ii) quantify aging in human cohorts, iii) provide a more robust index of the effects of private gene mutations and specific polymorphisms on aging and longevity, and iv) facilitate efficacy studies of therapies designed to promote healthy aging in humans. Following derivation of a composite BAS, prospective validation will be required to assess its accuracy in predicting development of disease, disability, and death. To that end, longitudinal research efforts such as the United States Precision Medicine Initiative seeking to enroll 1 million participants and the European MARK‐AGE Consortium with 3200 enrolled participants are seeking to define a set of human aging biomarkers (Burkle *et al*., 2015). Next‐generation sequencing, proteomics, and metabolomics show great promise to advance our understanding of complex biological processes. These technologies carry the potential to identify distinct aging signatures; however, they require additional investigation in epidemiologic studies prior to integration into a contemporary BAS. Although discovery of the fountain of youth remains elusive and unlikely, the outlook to characterize and mitigate age‐related morbidity is optimistic.

## Funding sources

NIH R01 HL51387 (DEV), NIH K08 HL128867 (BDS) and F32 HL129695 (SSK); and the Parker B. Francis Research Opportunity Award (BDS). The funding sources did not have any role in the preparation of this manuscript.

## Conflict of interests

The authors have no conflicts of interest to declare.
